# IPIAD- an augmentation regimen added to standard treatment of pancreatic ductal adenocarcinoma using already-marketed repurposed drugs irbesartan, pyrimethamine, itraconazole, azithromycin, and dapsone

**DOI:** 10.18632/oncoscience.594

**Published:** 2024-02-07

**Authors:** Richard E. Kast

**Affiliations:** ^1^IIAIGC Study Center, Burlington, VT 05401, USA

**Keywords:** gemcitabine, multidrug, paclitaxel, pancreatic ductal adenocarcinoma, repurposed

## Abstract

This short note presents the data and rationale for adding five generic non-oncology drugs from general medical practice to gemcitabine, nab-paclitaxel, a current standard cytotoxic chemotherapy of pancreatic ductal adenocarcinoma. The regimen, called IPIAD, uses an angiotensin receptor blocker (ARB) irbesartan indicated for treating hypertension, an old antimicrobial drug pyrimethamine indicated for treating toxoplasmosis or malaria, an old antifungal drug itraconazole, an old broad spectrum antibiotic azithromycin and an old antibiotic dapsone. In reviewing selected growth driving systems active in pancreatic ductal adenocarcinoma then comparing these with detailed data on ancillary attributes of the IPIAD drugs, one can predict clinical benefit and slowing growth of pancreatic ductal adenocarcinoma by this augmentation regimen.

## INTRODUCTION

This paper presents the rationale for adding five already approved and marketed generic drugs from general medical practice to the current standard current first line chemotherapy for pancreatic ductal adenocarcinoma (PDAC). This adjuvant regimen, called by its acronym, IPIAD, uses an old angiotensin receptor blocker (ARB) irbesartan, an old antimicrobial drug pyrimethamine, an old antifungal drug itraconazole, an old antibiotic azithromycin, and an old antibiotic dapsone.

PDAC is a highly desmoplastic tumor where nonmalignant tumor stroma plays a more active symbiotic role in growth of the malignant cells than is seen in most other cancers. The nonmalignant cells of PDAC grow in a trophic and immunosuppressive tumor environment that has neutrophils, macrophages, fibroblasts, and a desmoplastic stroma rich in hyaluronan [1]. This stroma requires a separate treatment regimen from the malignant PDAC cells. Survival rates at 5 years are low. At presentation, half of all PDAC have already metastasized. For unclear reasons, incidence of PDAC has been steadily increasing at 0.5 to 1% per year [1].

The IPIAD regimen was designed specifically to improve standard current chemotherapies for metastatic PDAC particularly in cases where the benefits of resection is questionable. Pancreatectomy and even oligometastases resection can be offered to those achieving tumor reduction with chemotherapy. IPIAD, by inhibiting aspects of growth drive in PDAC as outlined here, was constructed to help current standard gemcitabine, nab-paclitaxel achieve this bulk reduction with consequent survival benefits. May reduce the local mass size, promote tumor downstaging, and increase the likelihood of resection. Patients presenting with inoperable metastatic PDAC who experience a major response after neoadjuvant treatment can occasionally be restaged, have an increased survival, and some may then have tumors small enough to resect [1–3].

The IPIAD drugs were chosen by searching for potentially constructive intersections between the pathophysiologic growth drive mechanisms used by PDAC with the primary or ancillary physiological effects of common, low side effect medicines used in general medical practice (non-oncology drugs) that would interfere with them. The primary consideration in selecting drugs included in IPIAD was their having a low side effect risk and good safety when used in general medical practice.

This is the process that led to several repurposed multidrug regimens for adjunctive treatment of cancer, mainly glioblastoma, examples listed in Table 1 [4–9]. In these papers we outlined the reasons why a paradigm shift to multidrug regimens will be required to significantly stop a metastasized deadly cancer like glioblastoma or PDAC and why repurposed older drugs from general medicine should comprise many of those drugs. These reasons are summarized in Table 2.

**Table 1:** List of previous multidrug adjuvant regimens from the IIAIGC study center using repurposed, older, non-oncology drugs mainly but not exclusively directed at treating glioblastoma

Regimen	Repurposed drugs used	References
CUSP9v3	Aprepitant, auranofin, captopril, celecoxib, disulfiram, *itraconazole*, minocycline, ritonavir, sertraline	[4]
OPALS	*Pyrimethamine*, cyproheptadine, *azithromycin*, loratadine, spironolactone.	[5]
EIS	*Itraconazole*, metformin, naproxen, pirfenidone, quetiapine, rifampin	[6]
MTZ	Minocycline, telmisartan, zoledronic acid	[7]
ADZT	Apremilast, *dapsone*, telmisartan, zonisamide	[8]
MDACT	celecoxib, *dapsone*, disulfiram, *itraconazole*, *pyrimethamine*, telmisartan	[9]

See the references for details on how each adjuvant drug interferes with malignant growth pathways. Omitted from listing here are standard cytotoxic drugs meant to be used with a given regimen. Only the repurposed drugs from general medicine are listed. Four of the drugs discussed in the context of treating glioblastoma in these works, dapsone, azithromycin, itraconazole, and pyrimethamine, are included in IPIAD. These are mentioned in italics. Telmisartan, mentioned in underlines, is an ARB marketed to treat hypertension that is in the same class as irbesartan.

**Table 2:** Eleven core reasons why a multidrug adjunctive regimen using already-marketed drugs will be needed to stop an aggressive, metastatic, currently deadly cancer like PDAC

#	Rationale for using repurposed medicines
1.	They are already approved for medical use.
2.	General practitioners are already familiar with their use.
3.	They are generally cheap and readily available worldwide.
4.	They have well-known and low risk of side effects
5.	We are constrained to treat today’s illness with today’s tools.
6.	Directly cytotoxic, genotoxic drugs have limits on how many and how much they can be used.
7.	Cancer cells pathologically engage the same physiological systems as used by non-malignant tissues. Many drugs have already been developed for those core systems.
**#**	**Rationale for requirement of a multidrug regimen**
8.	Many drugs needed to address malignant cells’ shifting reliance on multiple alternate growth drives.
9.	Inherently, multiple malignant subpopulations exist in PDAC, each with its own set of growth drives and inhibition susceptibilities.
10.	Growth drives change over time, driven in part by treatments.
11.	Tumor stroma contributes to malignant growth and must be addressed separately.

Table 2 here assumes absence of a “silver bullet” that might target a simple underlying cause for the many physiological changes in malignant growth.

The recent pilot clinical study of one such multidrug regimen for glioblastoma, CUSP9v3, showed that ten drugs to treat this cancer can be given daily over many years with benefit and without problems if close patient follow up is in place and a low-risk drug mix was carefully chosen [4].

As of this writing in 2023, widely metastatic PDAC is usually incurable [10]. Small PDACs, caught and removed early, before metastasis, have a good prognosis, a 5 yr. Overall survival of >80% [11]. Thus current efforts are aimed at reducing the extent of PDAC tumor burden with pre-surgical chemotherapy and/or irradiation [12, 13].

Current chemotherapies for PDAC commonly use either a) gemcitabine, nab-paclitaxel or b) FOLFIRINOX (folinic acid, fluorouracil, irinotecan, oxaliplatin) [14]. IPIAD is designed to contribute to tumor mass downsizing, to be used alongside gemcitabine, nab-paclitaxel.

Below, the attributes of irbesartan, pyrimethamine, itraconazole, azithromycin, and dapsone are reviewed with focus on how these attributes interact with several established PDAC growth driving systems in a way that is predicted to meet our goals of tumor mass downsizing. Four of the IPIAD drugs, pyrimethamine, itraconazole, azithromycin, and dapsone have been discussed in the context of adjunctive treatment of glioblastoma, shown in Table 1.

### Irbesartan

#### The drug

Irbesartan is an ARB that binds to the angiotensin II receptor 1, preventing receptor activation by angiotensin II. Irbesartan is commonly used to treat hypertension [15, 16]. Side effects are minimal and usually well tolerated. Angiotensin converting enzyme inhibitors (ACEi) are a related older class of medicine for treating hypertension by reducing conversion of angiotensin I to angiotensin II.

#### Intersection with PDAC

A strong rationale for adding irbesartan to gemcitabine, nab-paclitaxel are four epidemiological studies:

1. The empirical data of Zhou et al. showed longer median survival in advanced PDAC in people who took irbesartan, ~15 months, alongside of gemcitabine, nab-paclitaxel, compared to those on gemcitabine, nab-paclitaxel alone, ~10 months [17]. That comparative study was done on the basis of preclinical study showing that PDAC with low expression of c-Jun responded better to gemcitabine compared to PDACs with high c-Jun expression [17] - that plus demonstrations that irbesartan lowered PDAC expression of c-Jun. It is noteworthy in this context of c-Jun and gemcitabine, that a 2015 study showed that PDAC survival prolongation by ARB use was particularly seen in those on a gemcitabine containing regimen [18, 19].
2. A 2023 study using a different cohort of 700 PDAC cases showed an association of angiotensin II signaling inhibition by ARBs or ACEi and longer survival compared to those not using an ARB or ACEi [20].
3. The epidemiological study of Keith et al. looking at 8,000 PDAC cases of all stages. They found a reduction in PDAC mortality in ARB users and some but lesser benefit from ACEi use [21].
4. Cerullo et al. studied records of 4299 PDAC cases who had resection for localized PDAC. Those on an ARB had a 24% reduced risk of death at 5 years [22].

A 2013 clinical trial in advanced metastatic PDAC using an ARB similar to irbesartan, candesartan 16 mg/day, plus standard gemcitabine gave a progression free survival (PFS) and overall survival that the authors considered similar to historical controls [23]. However, PFS was 3.5 months in those receiving 8 mg candesartan/day and 4.6 months in those receiving 16 mg/day [23]. Does this mean using the maximum recommended dose of candesartan, 32 mg/day, will give incremental benefit? Cf. the orthopedics aphorism “if a little force doesn’t work, maybe more force will”. Since PDAC tumors synthesize angiotensin II without use of ACE [24] this may account for the stronger PDAC growth inhibition seen with ARBs compared to ACEi.

Hypotension is a risk with any ARB use but can be frequently addressed in normotensives by some volume expansion with intake of a liter of salty tomato juice like V-8 juice™ or dose reduction.

Multiple animal studies showing ARB use inhibited PDAC growth preceded clinical ARB studies in PDAC [25–28].

One epidemiological study failed to find an association between ARB use and survival in PDAC [29].

Recognition that angiotensin II signaling forms one of the many overactive signaling systems active in glioblastoma led to inclusion of an angiotensin converting enzyme inhibitor in the CUSP9v3 regimen [4]. Many organs, including pancreas, have endogenous renin-angiotensin systems functioning independently from the renal-hepatic-lung system associated with hypertension [30]. The renin-angiotensin signaling system is often found deranged or hijacked to promote malignant growth across the common human cancers [31].

A further and important reason to add an ARB like irbesartan to PDAC treatment should be noted. The presence of sarcopenia is common and lowers survival duration in PDAC [32, 33]. Angiotensin II signaling at angiotensin II receptor 1, the receptor that ARBs block, contributes to sarcopenia across several diseases including heart failure, Alzheimer’s disease, and the common cancers [34, 35]. Several sartan class ARBs lowered muscle loss in patients with cardiovascular disease [36], in hemodialysis patients [37], and in old mice [38].

### Pyrimethamine

#### The drug

**Figure 1:**
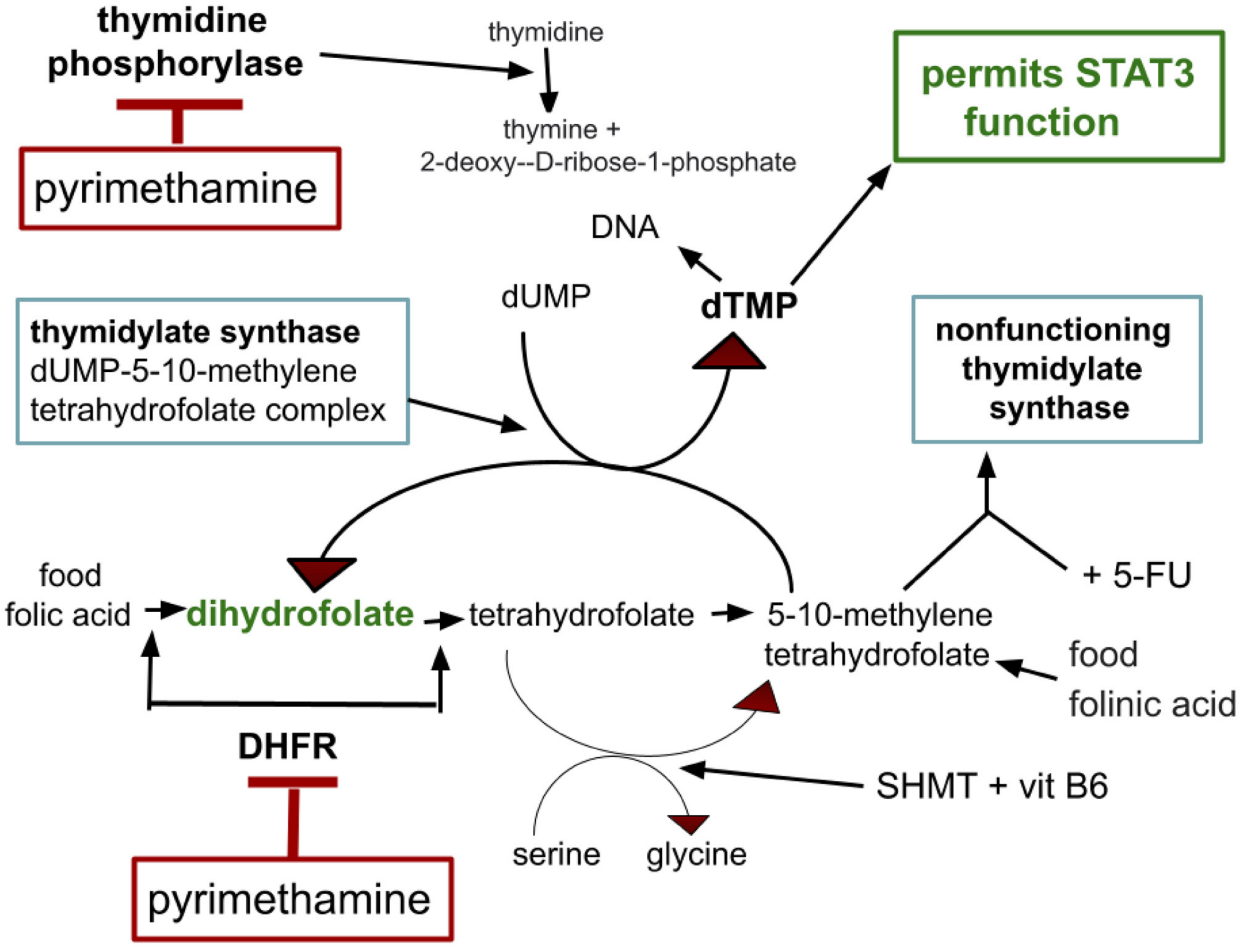
Simplified overview of pyrimethamine effects on the folate cycle and STAT3. DHFR, dihydromethylfolate transferase; 5-FU, fluorouracil; SHMT, serine hydroxymethyltransferase; vit B6, vitamin B6, (pyridoxal vitamers).

Pyrimethamine is an old generic antibiotic used today mainly in treatment or prophylaxis against Toxoplasma gondii, Pneumocystis jirovecii, Plasmodia and other protists. Pyrimethamine is one of several antibiotics that are based on dihydrofolate reductase (DHFR) inhibition. It is a lipophilic DHFR similar to hydrophilic methotrexate. Pyrimethamine also inhibits thymidine phosphorylase, methotrexate does not. How these inhibitions fit into folate metabolism is depicted in Figure 1.

#### Intersection with PDAC

Pyrimethamine has inhibitory effects on four growth drive elements of PDAC - (1) DHFR, (2) myeloid derived suppressor cells (MDSC), (3) STAT3, and (4) thymidine phosphorylase.

#### DHFR

DHFR is essential for normal functioning of the folate cycle. Pyrimethamine’s antibiotic activity in treating malaria, toxoplasmosis, pneumocystis, etc is based on a preferential inhibition of microbial DHFR compared to human but it does also inhibit human DHFR [39, 40]. Methotrexate is a standard DHFR inhibitor used in several cancer chemotherapies [41–43].

Pyrimethamine’s Ki = 38 nM at DHFR, is comparable to that of the more commonly used DHFR inhibitor methotrexate, Ki = 2.3 nM or the DHFR natural substrate folinic acid Ki = 320 nM, and folic acid Ki = 830 nM [39, 40]. For methotrexate to be active, it must be retained within the cell, which occurs when it becomes polyglutamated. Pyrimethamine does not require polyglutamination to be retained within cells. Pyrimethamine treatment at higher doses can give a reversible bone marrow suppression, making periodic blood monitoring advisable [44, 45].

In an acute myelogenous leukemia model, pyrimethamine was more effective in inhibiting growth than was methotrexate. *In vitro* proliferation was reduced 2.5 fold at 0.1 μM and 12.7 fold at 0.5 μM [46]. Several patients with polycythaemia vera and with essential thrombocythemia were successfully controlled with pyrimethamine, reported in 1987 [47].

Steady state pyrimethamine plasma level is ~1 μM at about 2–3 weeks following a dose of 12.5 mg/day, compared to ~6 μM with 50 mg/day [48]. Pyrimethamine has exhibited significant *in vitro* cytotoxicity to chronic lymphocytic leukemia cells within this dose range while showing no cytotoxicity to mixed leukocytes from healthy normals [48]. It is unclear why early reports in the 1970s of successful pyrimethamine treatment (2 mg/kg/day for 7 days) of meningeal recurrence of acute lymphoblastic leukemia in children have not been followed up [49].

#### MDSC and STAT3

Morphologically MDSC appear on H&E as neutrophils or monocytes. Neutrophil MDSC are CD15+ CD14− CD33dim HLA-DRneg. Monocyte MDSC are CD15− CD14 + CD33pos HLA-DRneg [50]. Although MDSC function is important in immune response moderation and normal wound repair, MDSC exert both immune response inhibiting and tumor growth trophic effects in the common cancers, including in PDAC [51–53].

STAT3 is a ~800 amino acid, 92 kDa transcription factor resident in an inactive form in cytoplasm under basal conditions. It is a member of a family of seven closely related transcription factors that are essential for normal cells’ function but often elevated and overactive in the common cancers, including in PDAC [54–56].

STAT3 is a signaling hub protein, a meeting point of many cellular signaling pathways, activated (phosphorylated) via many different inputs from these pathways. Several different cytokines acting on the outer cell membrane are transduced through the STAT3 signalling hub. Once activated (phosphorylated), P-STAT3 translocates to the nucleus as a dimer, where it binds to 9 base pair regions in the promoters of target genes to regulate transcription. In addition to regulating genes controlling proliferation, survival, and pluripotency, increase in P-STAT3 activity is a feature of inflammation [57–59]. P-STAT3 signaling plays a central role in MDSC survival and the production of immunoinhibiting arginase in MDSCs [60]. Elevation of P-STAT3 function drives increasing MDSC in PDAC [61–64].

MDSC synthesize and secrete arginase with consequent local arginine depletion inhibits T cell function [51, 65, 66]. Local arginase is just one of several T cell suppressing factors active in PDAC [53, 67, 68].

STAT3 is activated in PDAC intratumoral MDSC. Inhibiting STAT3 function reduces MDSC’s immune suppressing activity [69–72]. Inhibition of STAT3 with pyrimethamine in a murine breast cancer model resulted in decreased *in vivo* tumor growth and enhanced immune response [73]. Pyrimethamine similarly inhibited STAT3 and mesothelioma cell growth while having minimal effect on non-malignant pleural cell growth [74].

P-STAT3 activity is but one of the coalition of drivers increasing MDSC [75–77]. Inhibition of P-STAT3 function generally reduces the numbers and function of MDSC [78–80]. A study demonstrated a pyrimethamine mediated inhibition of STAT3 and proliferation of lung adenocarcinoma cells [81]. It has yet to be shown if the specific P-STAT3 function inhibition produced by pyrimethamine in clinical treatment of PDAC does this also or not.

#### Thymidine phosphorylase

Thymidine phosphorylase catalyzes phosphorylation of thymidine or deoxyuridine to respectively thymine or uracil and 2-deoxyribose 1-phosphate as in Figure 1. This is an essential step in thymidine recycling.

Thymidine phosphorylase activity is commonly increased in malignant tissue across the common cancers [82–84] and is specifically so in PDAC where increased thymidine phosphorylase levels correlated with shorter survival [85–89]. Thymidine phosphorylase is indeed a worthwhile target to inhibit in PDAC.

### Itraconazole

#### The drug

Itraconazole is a commonly used antifungal drug used to treat both minor skin infections with Tinea, Candida, Malassezia spp., etc., or more serious invasive fungal disease. Itraconazole is one of the CUSP9v3 drugs used in glioblastoma treatment and is seeing increasing use as adjunct in other cancers’ chemotherapies [4, 90]. By blocking fungal ergosterol synthesis, itraconazole disrupts fungal cell wall integrity resulting in fungal cell death [91].

A major problem in interpreting clinical data on itraconazole in PDAC is its poor and erratic absorption. Itraconazole must be given with 200–300 ml of a low pH liquid - Coke™, orange juice, or 15 ml of vinegar 5% acetic acid in 200 ml water are examples. It has been common to prescribe itraconazole without mentioning this essential aid to itraconazole’s absorption.

#### Preclinical data

Older reviews outlined several ways itraconazole interferes with cancer growth [3, 92, 93].

A Bak-1 activation dependent apoptosis was identified in CFPAC-1 cells. These data suggested that itraconazole exhibited antiproliferative effects in PDAC by inducing apoptosis through Bak-1 activation. [94]. Itraconazole induced PDAC cells’ cytotoxicity *in vitro* that could be reversed by recombinant TGF-beta [95].

#### Hedgehog

Itraconazole inhibits Hedgehog signaling (Hh) [96–99]. Hh signaling is a core signalling element in normal organogenesis [100–103]. Excessive Hh signaling is an important growth driver in PDAC [104–107] as it is in many of the common deadly cancers [108–110].

#### Antimicrobial function

Since the pancreatic duct has direct communication with the duodenum at the major duodenal papilla, and PDAC usually starts a few cm distal to that, it is not surprising that PDAC tissue is not normally sterile [111]. Retrograde travel deep into the pancreatic tree would be easy and has been shown to occur. Bacterial DNA was detected in 76% of resected PDACs and in 15% of normal pancreases [112]. Akut et al. found PDAC tissue had ~3000-fold increased fungal DNA compared to normal pancreases [113]. Malassezia, Pseudoxanthomonas, Streptomyces, Saccharopolyspora and Bacillus clausii, Porphyromonas gingivalis and Fusobacterium nucleatum can be found within PDAC resected tissue [111–116].

Details of the relationship between microbial pancreas colonization and PDAC development have not been worked out in detail but the risk/benefit would favor use of itraconazole (and azithromycin, vide infra) on the basis of such colonization and the evidence that such colonization is, in fact, an element promoting PDAC growth.

#### Empirical

A 2022 study of Sawasaki et al. treated 81 PDAC cases with 400 mg itraconazole/day for four days every 14 day cycle. The authors felt this resulted in longer survival compared to historical controls but this report lacked important details, preventing further interpretation [117].

A similar study in 2015 reported 38 advanced PDAC cases given docetaxel, gemcitabine, and carboplatin one day every 14 days with itraconazole 400 mg/d on days −2 to +2 from that. The authors felt this resulted in longer survival compared to historical controls but this report too lacked details that would allow further interpretation [93].

Both these studies were seriously flawed and lacked a true control group. The dose of itraconazole in these studies was needlessly low and the ten days of no itraconazole between dose days not justified by the ferocity of the disease they aimed to treat or by the pharmacokinetics of itraconazole.

Since itraconazole disrupts function of mammalian focal adhesion kinase with consequent decreased cell motility and angiogenesis [118], plus Hh and the other growth elements itraconazole inhibits, it should be given at high doses (as tolerated) and daily without interruption, even when the cytotoxic chemotherapy is given on an intermittent schedule.

A dramatic single case report was published in 2015 of an advanced unresectable PDAC in a 64 y/o man, stage III, T4, N1, M0. His CA19-9 level was 189 units/ml (0–37 units/ml). He was given capecitabine, and two cycles of cisplatin. Then, after restaging he was still unresectable. Palliative gemcitabine and erlotinib was started but stopped after a month when pulmonary histoplasmosis was diagnosed. After a six month course of itraconazole 200 mg/day a repeat PET/CT showed a much reduced tumor size that was deemed resectable, A Whipple procedure was done. Five years later repeat evaluations showed no evidence of tumor [119]. Single case reports are not proof of anything but neither should they be ignored.

### Azithromycin

#### The drug

Azithromycin is a broad-spectrum antibiotic that also has activity against several protists. Mechanism of action is by binding to microbial ribosomal RNA, stopping protein synthesis. It has a serum half-life of several days. Intracellular azithromycin levels are longer and are many times greater than serum levels. Neutrophil intracellular levels are several hundred times greater than serum levels [120]. Inhibition of autophagosome activity, lysosome leakage, and limiting dysfunctional inflammation are other azithromycin attributes seemingly independent of its antimicrobial activity [121].

#### Intersection with PDAC

Recent papers collected past research on the potential of azithromycin to interfere with aspects of malignant cell growth [121, 122].

Azithromycin inhibited lysosomal movement along microtubules by binding to tubulin but shows minimal cytotoxicity *in vitro* to non-transformed cells [123]. Azithromycin shows no evidence of cytotoxicity when used as antibiotic.

#### Empirical

Heterotopic non-small cell lung cancer tumors grew slower in azithromycin treated mice [123]. *In vitro* exposure to azithromycin gave significant cell death in cell lines of breast, ovarian, lung, pancreatic, and prostate cancers, and in glioblastoma and melanoma cell lines [124]. In another study of several lung cancer cell lines, azithromycin addition increased growth suppression of tyrosine kinases inhibitors and enhanced DNA damaging drugs’ carboplatin, doxorubicin and etoposide, cytotoxic effects. Importantly, non-transformed cells in culture were unaffected by azithromycin [125]. In this study azithromycin’s effect was secondary to creation of lysosomal damage and leakage. Specifically in PDAC cell lines azithromycin showed minimal cell number reduction after 48 hours co-incubation but with gefitinib but cell number reduction was 50% after coincubation with azithromycin at between 5 and 15 microM [126]. Azithromycin augmented cationic amphiphile lansoprazole’s cytotoxicity to squamous cell carcinoma and lung cancer cell lines. Evidence pointed to defective autophagosome function as the mechanism of action [127].

#### Autophagy

Autophagy is the catabolic process that takes place when an autophagosome fuses with a lysosome. Contained within lysosomes are many different proteases, nucleases, glycosidases, lipases, phospholipases, phosphatases, peptidases and sulfatases. A lysosomal membrane proton pump transfers protons from cytosol to lysosome, keeping lysosomes’ pH ~5, the optimum pH for the hydrolases. Lysosomes function to conserve amino acids, allowing their recycling. Accordingly, growth of squamous cell carcinoma cell lines in an amino acid sufficient culture medium becomes poor in the presence of azithromycin but goes back to baseline in an azithromycin containing medium with increased amino acid content [128].

Across the common cancers lysosomes tend to be more permeable than that of non-malignant cells. Since the common DNA damaging cancer chemotherapy drugs are sequestered in lysosomes, that relatively greater permeability is one of the origins of these drugs’ relatively selective cytotoxicity to malignant cells.

Anything that damages PDAC’s lysosomal membrane integrity will enhance cytotoxicity to a wide variety of pharmaceutical agents. Preclinical study of lysosomal permeabilization by experimental drugs sensitized PDAC’s cytotoxicity to gemcitabine [129, 130], to the HER1 (EGFR)/HER2 tyrosine kinase inhibitor lapatinib [131] to Ca++ release from damaged lysosomes [132], to tumor necrosis factor related apoptosis-inducing ligand (TRAIL) [133], and ro 5-fluorouracil [134, 135].

As discussed in section 4. above, PDAC is usually found to be nonsterile [111–116]. Many different microbes have been identified growing in PDAC tissue. If this colonization is causative or contributory to PDAC growth, metastasis, or chemotherapy resistance, this would be another advantage for adding azithromycin to standard gemcitabine.

Among cancers, PDAC is relatively resistant to autophagy and to apoptosis. The relationship between apoptosis and autophagy is not simple. Cell death occurs with excess autophagy as well as with inadequate autophagy function [136, 137].

### Dapsone

#### The drug

Dapsone is one of the first of modern antibiotics, introduced in the 1930s to clinical practice. Used today as an antibiotic for treating Hansen’s disease, malaria, toxoplasmosis, and tuberculosis i.e., dapsone also has an interesting side effect of reducing neutrophil degranulation and chemotaxis. On that basis dapsone reduces neutrophil mediated tissue damage in diseases like the bullous pemphigus and the neutrophilic dermatoses [138–141]. Dapsone reduced IL-8 synthesis in several experimental settings [142, 143]. As a consequence of IL-8 reduction, dapsone can lower the neutrophil to lymphocyte ratio (NLR).

It is on these same bases of (a) limiting IL-8, (b) inhibiting neutrophil degranulation and (c) preventing neutrophil chemotaxis, that dapsone is used to treat the neutrophil mediated rash of EGFR inhibitors osimertinib and erlotinib [5, 144–146]. By virtue of these three attributes, dapsone is used to reduce neutrophil mediated lung damage in acute respiratory distress (ARDS) including the ARDS of COVID19 [147–149].

#### Neutrophils and NLR in PDAC

Neutrophils contain significant amounts of VEGF and other angiogenic factors, and they deliver these growth factors to tumors, including to PDAC [150–154].

Dozens of previous studies in PDAC have shown that survival becomes shorter as the neutrophil-to-lymphocyte ratio becomes higher. These studies were reviewed in 2021 [155]. Four new, independent studies reporting in 2023 have confirmed these past findings [156–159]. All these studies reflect the hundreds of studies showing this same association across all the common deadly cancers [160–163].

The tumor trophic function of neutrophils in PDAC [164–166] is reflected by a similar role in other common cancers [167–170]. PDAC both secrete into bloodstream and excrete into pancreatic juice significant amounts of IL-8 [171–179]. This IL-8 is trophic to both the malignant cells and their supportive stroma cells in PDAC.

The above data documents the established ability of dapsone to impede neutrophil accumulation in the neutrophilic dermatoses. By the same dapsone attributes, when carried over to PDAC, dapsone is projected to reduce the angiogenic, tumor trophic, and immunosuppressive functions of the IL-8 attracted tumor infiltrating and systemic neutrophils.

## DISCUSSION AND CONCLUSION

Use of any untested experimental treatment involves risks. In the case of IPIAD, although the individual drugs are known to be of fairly low risk in their non-oncology use, the combination of these five drugs has never been tested. Although no particular drug-drug interaction is foreseen based on our extensive data on their pharmacology, surprises can’t be excluded.

Because of the many unknowns in the early clinical use of such untested regimens like IPIAD, careful follow up and monitoring are required to detect any untoward events. This means easy access 24 hours a day, 7 days a week by telephone to the prescribing physician with weekly in person meetings with review of systems, laboratory blood work, and addressing tolerability are minimum requirements.

The target doses listed in Table 3 are at the high end of tolerability. This is required by the hardiness of PDAC. Metastatic PDAC will not be slowed by half-measures. Using these target doses also becomes safer when the monitoring recommendations are followed. Given these considerations altogether and the alternatives available in 2023, continuous daily IPIAD added to standard gemcitabine, nab-paclitaxel, nab-paclitaxel must be regarded as a conservative approach to metastatic PDAC.

**Table 3:** Suggested target doses for IPIAD drugs

Drug	Suggested dose^*^
Irbesartan	300 mg × 1 qd
Pyrimethamine	50 mg × 1 qd
Itraconazole	300 mg twice daily
Azithromycin	500 mg × 1 qd
Dapsone	100 mg × 1 qd

^*^All doses must be individually tailored to the specific patient. For safety a minimum of weekly 1 on 1 evaluation with the prescribing physician must be in place. Adding one drug at a time and gradual uptitration to target doses as tolerated over three weeks allows early recognition of problems or poor tolerance. Judging by these medicines’ use in general medical practice, serious adverse events are expected to be rare. These doses are at the high end of their range, but an aggressive disease like PDAC requires and justifies this.

The reliability of relentless disease progression and short survival duration in metastatic PDAC plus our current lack of effective treatment to significantly lengthen survival, combined with the projected benign nature of continuous daily use of robust doses of the IPIAD drugs, altogether, warrant a pilot study of this regimen to run concomitantly with current standard cytotoxic treatments.

## Abbreviations

ACEi: angiotensin converting enzyme inhibitor
ARB: angiotensin receptor blocker
DHFR: dihydrofolate reductase
Hh: Hedgehog signaling
PDAC: pancreatic ductal adenocarcinoma
MDSC: myeloid derived suppressor cells
NLR: neutrophil to lymphocyte ratio
